# A New Family of Diverse Skin Peptides from the Microhylid Frog Genus *Phrynomantis*

**DOI:** 10.3390/molecules25040912

**Published:** 2020-02-18

**Authors:** Constantijn Raaymakers, Benoit Stijlemans, Charlotte Martin, Shabnam Zaman, Steven Ballet, An Martel, Frank Pasmans, Kim Roelants

**Affiliations:** 1 Amphibian Evolution Lab, Biology Department, Vrije Universiteit Brussel, Pleinlaan 2, 1050 Elsene, Belgium; tijnraaijmakers90@hotmail.com (C.R.); shabnam.zaman@vub.be (S.Z.); 2 Wildlife Health Ghent, Faculty of Veterinary Medicine, Ghent University, Salisburylaan 133, 9820 Merelbeke, Belgium; an.martel@ugent.be (A.M.); frank.pasmans@UGent.be (F.P.); 3 Unit of Cellular and Molecular Immunology, Vrije Universiteit Brussel, Pleinlaan 2, 1050 Elsene, Belgium; benoit.stijlemans@vub.be; 4 Myeloid Cell Immunology Lab, VIB Centre for Inflammation Research, Vrije Universiteit Brussel, Pleinlaan 2, 1050 Elsene, Belgium; 5 Research Group of Organic Chemistry, Department of Chemistry and Department of Bio-engineering Sciences, Vrije Universiteit Brussel, Pleinlaan 2, 1050 Elsene, Belgium; charlotte.martin@vub.be (C.M.); steven.ballet@vub.be (S.B.)

**Keywords:** amphibians, frogs, Microhylidae, poison, peptides, evolution

## Abstract

A wide range of frogs produce skin poisons composed of bioactive peptides for defence against pathogens, parasites and predators. While several frog families have been thoroughly screened for skin-secreted peptides, others, like the Microhylidae, have remained mostly unexplored. Previous studies of microhylids found no evidence of peptide secretion, suggesting that this defence adaptation was evolutionarily lost. We conducted transcriptome analyses of the skins of *Phrynomantis bifasciatus* and *Phrynomantis microps,* two African microhylid species long suspected to be poisonous. Our analyses reveal 17 evolutionary related transcripts that diversified from to those of cytolytic peptides found in other frog families. The 19 peptides predicted to be processed from these transcripts, named phrynomantins, show a striking structural diversity that is distinct from any previously identified frog skin peptide. Functional analyses of five phrynomantins confirm the loss of a cytolytic function and the absence of insecticidal or proinflammatory activity, suggesting that they represent an evolutionary transition to a new, yet unknown function. Our study shows that peptides have been retained in the defence poison of at least one microhylid lineage and encourages research on similarly understudied taxa to further elucidate the diversity and evolution of skin defence molecules.

## 1. Introduction

The amphibian skin is a delicate but sophisticated organ that is directly exposed to the challenges of the animal’s environment. To ward off harmful microorganisms, or to prevent becoming a predator’s next meal, a wide range of frogs (Anura) secrete a mixture of antimicrobial peptides and toxins from poison glands in their skin [1,2,3]. Over the past decades, these bioactive peptides have been predominantly studied from a pharmacological perspective, focussing on their potential medical application in wound healing [4,5], regulating glucose metabolism [6,7] and acting as broad-spectrum antibiotics [8,9]. Although the presence of skin peptides has been described in 13 anuran families [1,2,10], only five lineages appear to have received the great majority of scientific attention. Besides the genera *Xenopus* (Pipidae) and *Bombina* (Bombinatoridae), a limited number of genera in the families Phyllomedusidae (*Agalychnis* and *Phyllomedusa*), Pelodryadidae (*Litoria*) and Ranidae (*Rana*, *Lithobates* and *Odorrana*) have been particularly thoroughly explored for the presence of therapeutically promising peptides [11,12,13]. These peptides often display different bioactivities and range from antimicrobial or antioxidant agents, to hormone analogues that can cause sickness, hypotension or inflammation [1,2]. Despite their structural and functional differences, most of these skin peptides are posttranslationally cleaved from evolutionary related (homologous) precursor proteins that descended from a single ancestral gene in an early neobatrachian (advanced) frog an estimated 150 million years ago [11,12,14,15]. Related precursor proteins have been described in species of the frog families Leptodactylidae, Hyperoliidae, Dicroglossidae and Rhacophoridae, indicating that the same molecular defence system has been evolutionary preserved in several less explored neobatrachian families [2,15]. These precursor proteins have been classified in the ‘Frog Skin Active Peptide’ (FSAP) superfamily in the Uniprot database [16,17] and comprises a multitude of different peptide families defined on the basis of their taxonomic occurrence and/or sequence similarity (including brevinins, temporins, esculentins, caerins and dermaseptins and many others). FSAP precursor proteins are typically between 60 and 200 amino acids long and contain an evolutionary conserved *N*-terminal signal peptide and one or several acidic spacer regions (similar to vertebrate prepro-hormone precursors) that are proteolytically cleaved from the *C*-terminal bioactive peptide [1,12,14]. Although the past two decades have seen a drastic increase in our knowledge of the FSAP superfamily [1,2,11,13], the focus on a select number of frog taxa has left the skin secretions of a large diversity of neobatrachian frogs unexplored.

One such poorly explored taxon is the family Microhylidae (narrow-mouthed frogs). Microhylidae represent one of the largest and most ecologically diverse frog families, with well over 600 recognised species in 13 subfamilies distributed across the Americas, Asia, Africa, Madagascar and the Australo-Papuan region [18,19]. Despite this diversity, the study of skin secretion has been limited to a handful of species [1,20]. A toxinological analysis of the South American species *Dermatonotus muelleri* (Gastrophryninae) revealed high concentrations of the amino acid tryptophan [21], while for the Madagasacan *Dyscophus guineti* (Dyscophinae) and the Asian *Kaloula pulchra* (Microhylinae), only serine protease inhibitor proteins were identified [22,23]. None of these species were found to secrete peptides. However, peptides of the FSAP superfamily may have been preserved in the African genus *Phrynomantis* (Phrynomerinae). Species of this genus are conspicuously coloured and at least two of them, *Phrynomantis microps* and *Phrynomantis bifasciatus*, are known to be toxic [24,25]. In addition, two peptides have been described from the skin secretion of *P*. *microps* [20]. During dry periods, this species is known to take shelter in the nests of *Paltothyreus tarsatus*, an aggressive ant species that otherwise attacks any nest intruder [20,26]. The two identified peptides were shown to delay the aggressive behaviour of the ants, indicating that these compounds may be involved in a form of chemical camouflage or appeasement [20].

We conducted a transcriptome analysis to investigate the expression of peptide-encoding genes in the skins of the species *P. bifasciatus* and *P. microps*. Although the genetic basis of the two previously identified *P. microps* peptides remains undisclosed, our study shows that FSAP-related peptides have been retained in this microhylid lineage. Because of their highly derived sequences, we describe the identified peptides as a new family called phrynomantins.

## 2. Results

### 2.1. Skin Transcriptomes Elucidate the Diversity of Secretory Proteins in Phrynomantis

To obtain an overview of the genes expressed in the skin of the two *Phrynomantis* species, we extracted total RNA from dorsal skin. RNA-seq analysis of these samples (see Methods) yielded totals of 51.76 million and 30.58 million high-quality reads for *P. bifasciatus* and *P microps*, respectively. *De novo* transcript assembly using Trinity [27] and subsequent clustering of very similar contigs using CD-hit [28] resulted in 250,508 (*P. bifasciatus*) and 188,220 (*P. microps*) nonredundant transcript contigs for both species respectively. Subsequent mapping of reads against these transcript contigs reveal a similarly skewed expression profile for both libraries, in which only 759 transcript contigs (*P. bifasciatus*) and 780 transcript contigs (*P. microps*) have estimated expression levels above 100 transcripts per million (TPM). Screening of both transcript libraries using the Basic Local Alignment Search Tool (BLAST) of the Genbank database of the National Center for Biotechnology Information (NCBI) indicates that many of their highest expressed transcripts encode intracellular housekeeping genes (like ribosomal proteins and elongation factors) and extracellular structural protein genes (like keratins, tectorin-like proteins and mucins). In addition, BLAST searches against the same database as well as an in-house database of published amphibian secretory precursor proteins show that other highly expressed transcripts in *Phrynomantis* encode members of multiple protein families that are frequently encountered in frog skin secretions (Table 1). First, by far the highest expressed is the FSAP superfamily, accounting for ten full-length transcripts totalling 13,437 TPM in *P. bifasciatus* and seven full-length transcripts adding up to 12,637 TPM in *P. microps*. As the FSAP superfamily constitutes a major component of skin secretions in other frog lineages, their finding in *Phrynomantis* is a first indication that peptide secretion has been evolutionary preserved in at least one microhylid lineage. Second, our BLAST searches demonstrate the expression of at least five antimicrobial peptide/protein families that are known from a wide range of vertebrates (Table 1). While transcripts encoding beta-defensin, cathelicidin and G-type lysozyme are expressed at low levels (all <10 TPM), bactericidal permeability-increasing protein (BPI) and C-type lysozyme transcripts are highly expressed (>100 TPM). Third, a large number of transcripts encode small proteins with cysteine motifs (domains) that characterise five different families of serine protease inhibitors as defined in the Uniprot and PFAM databases [17,29]. These include Kazal-type serine protease inhibitor domain (protein family PF00050), kunitz/bovine pancreatic trypsin inhibitor (BPTI) domain (PF00014), serpin-type domain (PF00079), trypsin-inhibitor like (TIL) cysteine rich domain (PF01826) and whey acidic protein-type (WAP) ‘four-disulphide core’ domain (PF00095). The combined expression values of these transcripts indicate that serine protease inhibitors are a major functional class of proteins in the skins of both *Phrynomantis* species (Table 1). Fourth, BLAST searches did not reveal any transcripts that encode apparent hormone-derived toxins as found in other amphibians, like bombesins, caeruleins or prokineticins. However, transcripts encoding the hormones gastrin, glucagon, bradykinin, natriuretic peptide, neurotensin were recovered at very low expression levels (<10 TPM). One exception is secretogranin, a common constituent of vertebrate endocrine gland granules involved in granule biogenesis and acting as precursor protein of several subsequently cleaved hormone peptides [30]. Finally, we did not identify any transcript encoding a candidate precursor protein of either of the two previously described *P. microps* peptides [20]. Apart from blastx searches, a text screen of the peptide sequences obtained by translating all open-reading frames above 150 base pairs in our libraries did not reveal either one of the peptides’ sequences.

### 2.2. Phrynomantins: The First Microhylid Representatives of the FSAP Superfamily

**Figure 1:**
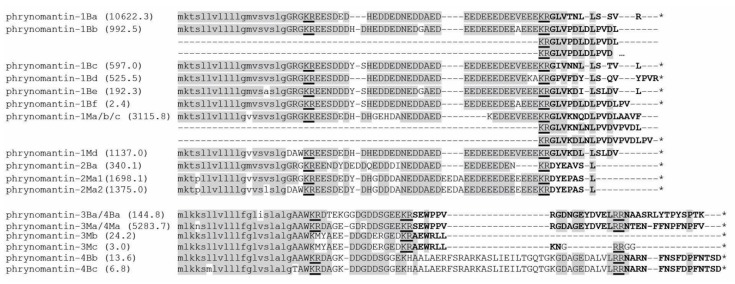
Sequence alignment of the 17 phrynomantin precursor proteins encoded by *Phrynomantis* skin transcripts. Predicted *N*-terminal signal peptides are printed in lower case, putative cleavage sites are underlined, predicted functional peptides (19 in total) are bold-faced. Conserved residues (identical in ≥50% of aligned sequences) are shaded in grey. Numbers behind sequences represent expression estimates (in transcripts per million; TPM).

The recovered FSAP transcripts encode precursor proteins between 44 and 110 amino-acids long, including an *N*-terminal signal peptide, an acidic spacer that typifies proteins of the FSAP superfamily and one or several lysine-arginine and arginine-arginine motifs corresponding to common peptidase cleavage sites (Figure 1). Sequence alignments show that two types of precursor proteins can be distinguished in both *Phrynomantis* species, mainly differing by variation in the signal peptide sequences and length of their acidic spacers.

**Figure 2:**
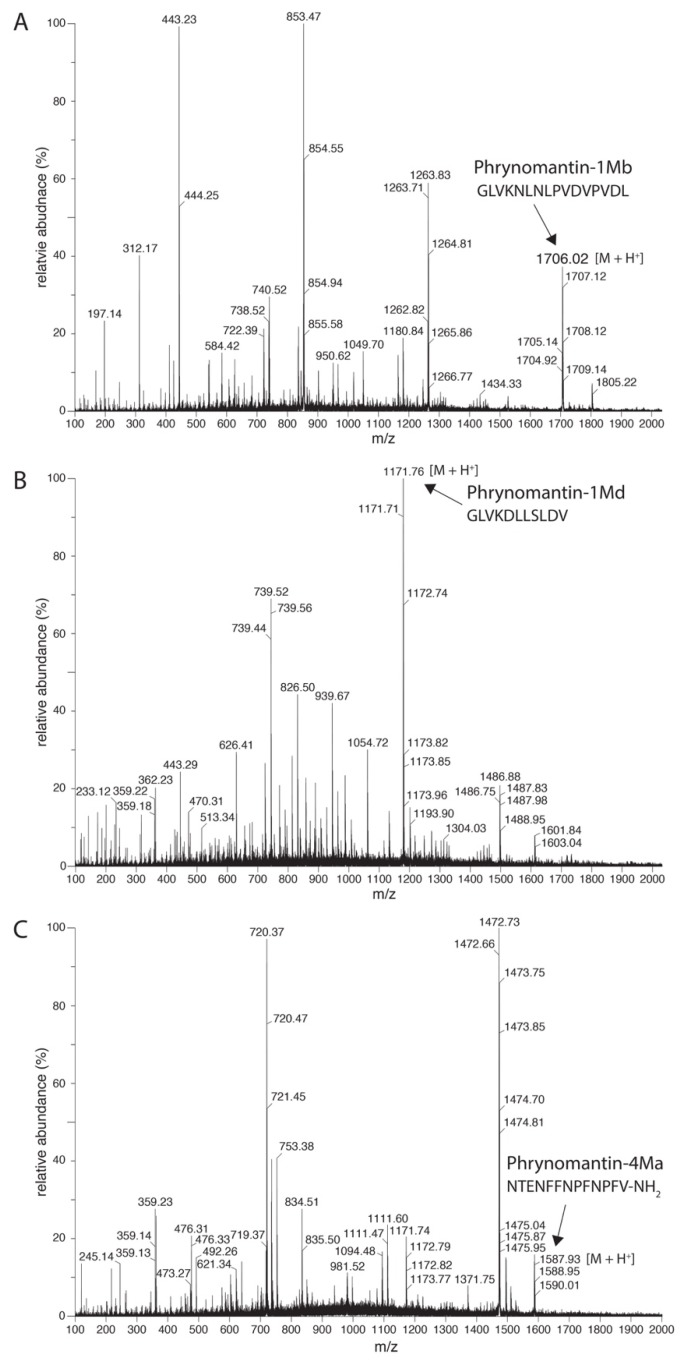
Confirmation of phrynomantin secretion by *P. microps* using liquid chromatography-mass spectrometry (LC-MS). Mass spectrometry data obtained by ESI+ identify phrynomantin-1Mb (**A**), phrynomantin-1Md (**B**) and phrynomantin-4Ma (**C**) in skin secretion samples with the peptide sequence shown above their respective ion peaks.

Based on putative cleavage sites, we predict the processing of at least ten different peptides from these precursor proteins in *P. bifasciatus* and nine peptides in *P. microps* (Figure 1, Table 2). Four precursor proteins seem to include two or three tandemly organised peptides, some of which are very similar or even identical to each other (e.g., phrynomantin-1Bb and -1Ma/b/c). One peptide, phrynomantin-2Ma, is processed from two different precursor proteins (phrynomantin-2Ma1 and -2Ma2). While the majority of FSAP in other frog taxa is cationic, 13 of the 19 predicted phrynomantins have a negative net charge (Table 2). In addition, most of them seem to lack the capacity to adopt an alpha-helical conformation, as predicted by the online prediction tool PsiPred [31]. Liquid chromatography-mass spectrometry (LC-MS) analysis of *P. microps* skin secretion confirmed the presence of three of its nine predicted peptides—phrynomantin-M1b, -M1d and -M4a (Figure 2).

### 2.3. Phrynomantins Serve a yet Unknown Function

Five *P. microps* peptides were *de novo* synthesised and tested for various bioactivities. These peptides included the three phrynomantins confirmed by LC-MS and were further selected to represent the highest expressed transcripts and broadly cover the sequence diversity of phrynomantins. Even at a high concentration (512 µM), none of these peptides showed cytolytic activity against the gram-negative bacterial species *Escherichia coli*, the gram-positive species *Staphylococcus aureus* or against Caco-2 human epithelial cells (data not shown).

**Figure 3:**
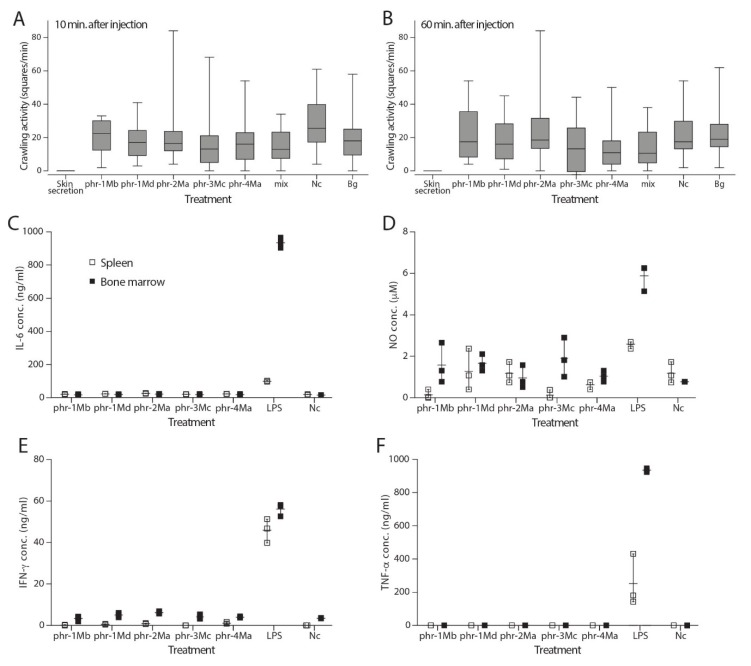
Assessing the insecticidal and inflammatory activity of *P. microps* skin secretion and its phrynomantin peptides. **A-B)** the effect of injecting 6× diluted skin secretion or peptide (20 µL at 1 mM) on crawling activity on wax moth larvae (n = 15-21) after 10 (**A**) and 60 min (**B**). **C-F**) Effect of phrynomantins (100 µM) on levels of IL-6 (**C**), NO (**D**), IFN-γ (**E**) and TNF-α (**F**) in cell medium of mouse spleen (white squares) and bone marrow (black squares) cell cultures (n = 3). Data in **A** and **B** are shown as boxplots and in **C-F** as individual data points, showing the range (vertical line) and average (horizontal line). Overlapping symbols might appear as single data points. Peptides are labelled as phr (phrynomantin) with their respective peptide number (see Table 2), Bg = background (no injection), Nc = negative control (injection of water), mix = mixture of all tested phrynomantins (each at 1 mM), LPS = *E. coli* endotoxin/lipopolysaccharide.

Braun et al. [26] mention a toxic effect of *P. microps* poison towards ants. To test whether newly discovered peptides could function as general insecticidal toxins, we performed toxicity experiments with the greater wax moth *Galleria mellonella*. Injection of *P. microps* skin secretion resulted in death of the wax moth larvae within one minute, showing a potent insecticidal effect. However, when individual peptides were injected, no reduced crawling activity was observed after either 10 min (Figure 3A; *X*^2^ = 8.12, df = 8, *p* = 0.422) or 60 min (Figure 3B; *X*^2^ = 5,16, df = 8, *p* = 0.740). Similarly, none of the peptides caused changes in larval body colouration or response to stimuli. Injecting the five peptides together did not have any significant effect either, indicating the absence of a synergistic activity between these peptides.

The skin secretion of *P. microps* has been described as causing an inflammatory response in humans [32]. We therefore tested the effect of the five phrynomantins on the production of proinflammatory cytokines by murine spleen and bone marrow cell cultures [33]. When treating these cell cultures with the peptides at concentrations of 100 µM, no change in proinflammatory cytokine secretion compared to a negative control was apparent (Figure 3C–F). On the contrary, treating immune cells with bacterial LPS resulted in an expected increase in the secretion of TNF-α, IFN-γ, NO and IL-6 after 24 h, indicating that our experimental setup worked correctly.

### 2.4. Evolutionary Origin and Diversification of Phrynomantins

**Figure 4:**
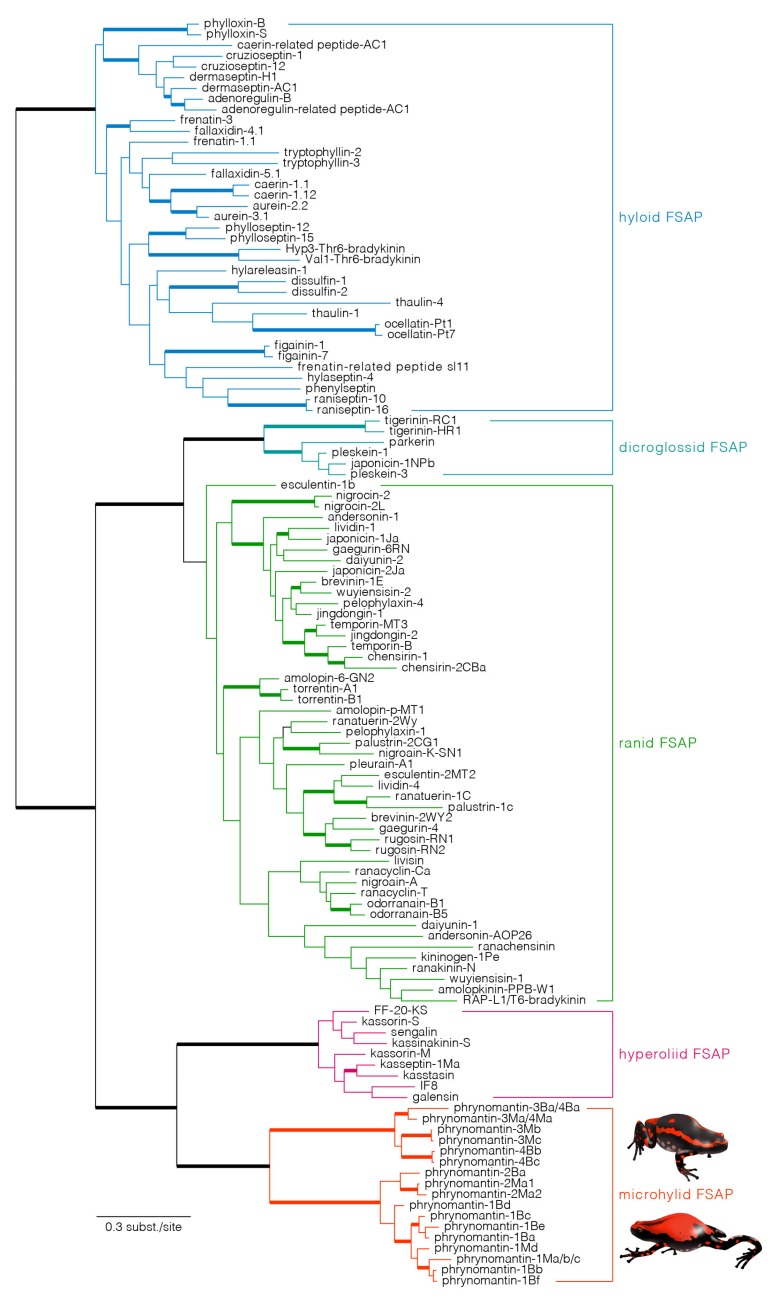
The evolutionary diversification of frog skin active peptide (FSAP) genes in Neobatrachia (advanced frogs). The depicted tree is a Bayesian consensus phylogram based on two parallel Markov chain Monte Carlo runs of 20 million generations each, using a GTR + G + I model of nucleotide substitution. Branches with posterior probabilities ≥ 0.95 are drawn as thick lines. Each clade comprising all FSAP representatives of a single frog family is highlighted in a different colour; the clade composed of phrynomantins is highlighted in red.

Phylogenetic reconstruction of FSAP superfamily evolution is *a priori* unrooted since it is impossible to assign a reliable outgroup to the dataset (i.e., homologous sequences for which a basal divergence with FSAP sequences is certain). However, by applying the maximum parsimony principle to gene duplication and loss (i.e., minimising the number of duplication events), the tree can be rooted such that it implies a single basal divergence between hyloid and ranoid sequences (Figure 4). The resulting rooted tree is largely compatible with current hypotheses regarding the phylogenetic relationships of major neobatrachian frog lineages [34,35,36]. Besides providing maximum support for the ranoid-hyloid bipartition, high Bayesian posterior probabilities were obtained for clades combining—(1) all dicroglossid sequences, (2) all dicroglossid + ranid sequences, (3) all *Kassina* sequences and (4) all phrynomantins. In addition, phrynomantins were recovered as the sister lineage of the *Kassina* clade, which is consistent with a closer relationship between Hyperoliidae (to which *Kassina* belongs) and Microhylidae (to which *Phrynomantis* belongs) [35,36]. Within the phrynomantin clade, two well-supported branches confirm our previous observation of the presence of two distinct precursor types, separating phrynomantins-1 and -2 from phrynomantins-3 and -4 (compare Figure 1 and Figure 4).

## 3. Discussion

We used transcriptomic analyses to obtain a comprehensive overview of the diversity of skin-secreted peptides and proteins in two species of *Phrynomantis*, a microhylid genus long suspected to be poisonous. Our data reveal both similarities and differences with the skin secretions of other frog taxa. A first similarity is the presence of serine protease inhibitors; the large diversity and high expression of multiple transcripts indicate that these constitute an important skin component in *Phrynomantis* species. The five families of serine protease inhibitors found in the skin of both species (Table 1) have been found before in other taxa, although typically as one or few closely related proteins [2,22,37,38,39,40]. Our results demonstrate that a frog’s skin repertoire of serine protease inhibitors may be extremely abundant and diverse, combining multiple structurally unrelated protein families. The exact function of these proteins remains unknown but it has been postulated that they inhibit the frog’s endogenous peptidases that release functional skin peptides from their precursors, thereby preventing the premature activation of toxins [2]. However, another plausible hypothesis is that a large abundance of protease inhibitors in skin secretion may protect cosecreted bioactive peptides against exogenous enzymatic degradation.

Second, we find a considerable diversity of transcripts encoding homologues of cytolytic antimicrobial peptides and proteins (AMPs). Cytolytic peptides are a widespread and well-documented component of amphibian skin secretions but typically constitute peptide families confined to a specific taxon or clade, like magainins (in pipid frogs), bombinins (in bombinatorid frogs) and members of the FSAP superfamily (in neobatrachian frogs) [1,2]. The primary role of these frog-specific peptides as antimicrobial agents of the innate immune system has recently been challenged in favor of an antipredatory function [1,15]. However, in both *Phrynomantis* species, AMP transcripts represent ancient protein families that are evolutionary conserved among vertebrates and whose role in innate immunity is undisputed. While beta-defensins, cathelicidins and lysozymes have been sporadically found in amphibians before [41,42,43], bactericidal permeability-increasing proteins (BPI) represent a new finding. Unlike other AMPs, they are large glycoproteins (typically 450-550 amino-acids) whose cytotoxic activity against bacteria is thought to be mediated by their affinity for negatively charged lipopolysaccharides [44]. It would be interesting to investigate whether the diversity of ancient vertebrate AMPs is similar across all frog taxa or whether it reflects an evolutionary pattern of functional replacement with frog-specific cytolytic peptides. At least in *Phrynomantis*, ancient vertebrate AMP families seem to serve the antimicrobial function that is attributed to frog-specific cytolytic peptides in other taxa.

Third, phrynomantins, like FSAP in other frog taxa, represent a major structural diversity indicative of fast sequence diversification. Although only three of the nine predicted *P. microps* peptides were confirmed by LC-MS, the high expression levels of their transcripts indicate that they represent an important component of the *Phrynomantis* skin secretion. Discrepancies between transcriptome and proteome data have been reported before (e.g., in snake venom studies) [45,46] and may be explained by biological and/or analytical factors. Biological factors may include uncorrelated temporal variation in transcription and peptide synthesis, posttranscriptional regulatory processes and rapid peptide degradation upon secretion. Analytical factors may include false positives in transcript-based peptide prediction, peptide concentrations below the LC-MS detection threshold, (in) stability of the charged peptides, possible co-elution of molecules and adduct formation complicating the interpretation of MS spectra.

Unlike most known FSAP, the majority of predicted phrynomantins is anionic. Although negatively charged skin peptides have been described in frogs before [2,47], cytolytic skin peptides are predominantly positively charged pore-forming peptides that are presumed to bind negatively charged constituents of the outer surface of cell membranes [1,2]. Contrary to phrynomantins, anionic peptides of the genera *Bombina*, *Xenopus* and *Leptodactylus* have been found to have cytolytic activity to some degree [47,48,49]. In the case of those peptides, the pore-forming effect is likely attributed to their high hydrophobic moment and helicity, which facilitate cell membrane binding and insertion. The absence or low degree of helicity in the majority of phrynomantins could explain their lack of cytolytic activity.

To test bioactivities other than cytotoxicity, we relied on the scarce published information on *P. microps* skin secretion [20,24,25,26]. Based on our results, we cannot conclude that any of these peptides functions as an inflammatory toxin or broad-spectrum insecticidal agent. However, since we only tested for a general toxic effect on a commonly used insect model, it is still possible that these peptides could have adverse effects on sympatric arthropods or even function as specific toxins against the *Paltothyreus tarsatus* ants whose nests are inhabited by *P. microps* frogs [20]. Nonetheless, the observation that injection of the frog’s full secretion shows an instant lethal effect on the wax moth larvae suggests that this insect model is suitable for evaluating toxic components in *Phrynomantis* poison. Neither individual phrynomantin peptide nor the mixture of five peptides produced any observable adverse effects on the larvae, indicating that another, yet unidentified component of the skin secretion is responsible for the observed insecticidal effect. Apart from peptides, many amphibians possess poisons based on steroidal toxins (like bufadienolides), alkaloids or bioactive amines (like serotonin) [50,51]. Additional chemical analysis should be conducted to verify whether such molecules also occur in *Phrynomantis* skin secretions. However, the consistently high expression of phrynomantins in both species suggests that they serve an important function for the frogs. Future studies that aim to characterise the biological role of phrynomantins could perhaps start from known bioactivities of noncytolytic peptides belonging to the FSAP superfamily, such as bradykinins, tachykinins or dermorphin.

One understudied effect that could increase a frog’s survival chance during a predator attack is a foul or bitter taste. The capacity to perceive bitter molecules is generally regarded as a widespread adaptation in animals to recognise and repel toxic food [52,53,54] and prey animals can take advantage of taste perception in predators by secreting bitter-tasting molecules. When carefully tasting small volumes of *P. microps* peptide solutions without ingestion, we noticed that two of them, phrynomantin-3Mc and phrynomantin-4Ma, indeed have a bitter taste. Phenylseptin, an FSAP from the secretion of the hylid tree frog *Boana punctata*, was previously found to elicit a bitter taste causing an adverse response in mice [55]. The bitterness of Phenylseptin was attributed to a repeat sequence of three phenylalanine residues. Together with other amino acid motifs (e.g.; Gly-Leu, Gly-Phe) Phe-repeats have been shown to facilitate bitter taste receptor activation [55,56]. Interestingly, phrynomantin-4Ma contains a Phe-Phe (FF) motif that could explain its bitter taste, while phrynomantin-M3a possesses a bitter taste despite the absence of such motif. Whether these peptides can truly protect a frog from predation via an adverse taste response could be explored via an integrative approach involving in vitro receptor activation assays and in vivo behavioural trials.

Despite their structural uniqueness and unknown bioactivity, phrynomantins represent the evolutionary counterpart of AMPs and neuropeptides found in other neobatrachian frogs. Our phylogenetic analysis recovers phrynomantins as closely related to the peptides described from the *Kassina* species [1,2], including mostly AMPs (e.g., kasseptins, galensin and senegalin) and the histamine-releasing peptide kassinakinin S [57]. This result mirrors the prevalent hypothesis on neobatrachian phylogeny [34,35,36]—the majority of molecular phylogenetic studies of frogs support a sister-clade relationship between Microhylidae (including *Phrynomantis*) and Afrobatrachia, an African clade including Hyperoliidae (and thus *Kassina*) along with Arthroleptidae, Brevicipitidae and Hemisotidae [35,36]. In addition, the inferred FSAP tree supports monophyly of phrynomantins, as well as of *Kassina* FSAP, ranid + dicroglossid FSAP and hyloid FSAP. This pattern of taxon-specific FSAP clades suggests an early neobatrachian origin of the superfamily but a much later onset of its expansion. Although a single FSAP gene appeared before the last common ancestor of hyloid and ranoid frogs (as previously shown) [14], it only underwent major diversification after the divergence of the major neobatrachian lineages, by multiple gene duplication events within each of these frog taxa.

In contrast to Ranidae and Hylidae, and despite a comparable ecological and taxonomic diversity, microhylid frogs have remained largely understudied when it comes to their skin secretion. Apart from *P. microps* [20], only three microhylid species have been the subject of published investigation—*Dermatonotus muelleri* (Gastrophryninae), *Dyscophus guineti* (Dyscophinae) and *Kaloula pulchra* (Microhylinae). Peptides were not recovered in any of these species, suggesting the evolutionary loss of peptide secretion and of the FSAP superfamily. The finding of FSAP members in *Phrynomantis* (Phrynomerinae) implies that this peptide superfamily has been preserved in at least one microhylid subfamily. Interestingly, although phylogenetic relationships among microhylid subfamilies remain largely unresolved, most recent molecular studies agree on a basal divergence of Phrynomerinae from all other microhylids [18,19,34,35,36,58,59]. The absence of FSAP across various microhylid taxa, along with their presence in *Phrynomantis*, is consistent with a single evolutionary loss of this peptide system in an early stage of the microhylid radiation, after the basal divergence of Phrynomerinae. Screening of microhylid frogs representing additional subfamilies could further elucidate the evolutionary history of this skin peptide repertoire.

Rather unexpectedly, our analyses did not recover any transcript that could encode either of the two previously described *P. microps* peptides reported to be involved in ant repellence [20]. Similarly, no trace of the peptides was found by subsequent peptidome analyses. Several factors could explain why these peptides are absent from our data. First, their absence could be attributed to geographic differences in peptide repertoires among different *P. microps* populations, since this species has a wide distribution range across the savannah regions of West Africa [20,25,26]. Such variation has been found across populations of North American and European ranid frogs [60,61] and may either involve sequence differences (different populations producing different isoforms of a single peptide family) or variation in peptide expression (peptides are produced in some populations but not in others). In *P. microps*, such variation may be correlated to the extent to which different populations aestivate in ant nests. Second, temporal differences in gene expression could explain why our captive animals seemed to lack the two chemical camouflage peptides. In *Pelophylax* frogs, peptide synthesis was found to be influenced by microbial factors, with animals kept under sterile conditions ceasing to produce AMPs. Rehousing of animals with a normal microbial community restored peptide synthesis in the skin [62,63], showing that environmental factors can impact the composition of amphibian skin secretions. It is therefore possible that *P. microps* only synthesises these molecules when living alongside *P. tarsatus* ants.

## 4. Materials and Methods

### 4.1. Ethics Statement

Experiments involving live animals were conducted in accordance with European guidelines and Belgian legislation on animal housing and experimentation and were approved by the Ethical Committee of Animal Experimentation of the Vrije Universiteit Brussel (Permit nos. EC15-AAA-2 and EC16-334-1).

### 4.2. Animal Housing and Sample Collection

Adult *Phrynomantis bifasciatus* (n = 2) and *Phrynomantis microps* frogs (n = 3) were purchased from terrarium shops. The *P. bifasciatus* frogs were immediately euthanized using a 10% lidocaine solution (10 µL/g body weight) and freshly dissected dorsal skin tissue was stored in RNAlater (Sigma Aldrich). After overnight cooling at 4 °C, the tissue samples were transferred to −20 °C for long-term storage. Skin samples of two *P. microps* frogs were obtained in the same way but after sampling of their skin secretion. The *P. microps* frogs were housed in an animalarium with a 12/12 h day-and-night cycle at 21–25 °C in glass terraria of 45 × 45 × 60cm (L × B × H). Skin secretion was sampled by placing each frog separately in a Ziploc bag and manually massaging them to simulate the secretion. After five minutes, secreted fluid on the frogs’ skin and the bags’ inner surface was collected in a microcentrifuge tube using a spatula. For peptidome analyses, the secretion of the three animals was pooled and diluted by adding five volumes of 1% (*V*/*V*) trifluoroacetic acid (TFA) and dissolved by brief vortexing. The pooled samples were filtered twice through a 0.45 µm pore filter (Acrodisc^®^, Pall Laboratories Co.; Port Washington, NY, USA) and stored at −20 °C. For insecticidal assays (see below), fresh secretion samples of the same three frogs were pooled and diluted with five volumes of ultrapure (milli-Q) water.

### 4.3. Transcriptome Analyses

Skin tissue (150 mg per individual frog) was homogenized using a GentleMacs^TM^ dissociator (Milteny Biotec; Bergisch Gladbach, Germany) and RNA was extracted using an RNeasy Universal Plus Midi kit (Qiagen; Hilden, Germany). Purified RNA of individual frogs was pooled together for RNA-seq analysis. A paired-end sequencing library was constructed using a TruSeq stranded RNA-seq library preparation kit (Illumina Inc., San Diego, CA, USA) and the Illumina HiSeq^TM^ 2500 sequencing system (outsourced to BaseClear BV, Leiden, The Netherlands). After quality control (FASTQC v0.10.0 plus in-house QC protocols), results were further analysed using the Illumina Casava pipeline (v.1.8.3) to generate FASTQ sequence files. Transcript sequences were reconstructed *de novo* using a pipeline involving contig assembly by Trinity 2.5.1 [27], clustering of closely related contigs using CD-Hit EST [28] and estimation of transcript expression levels (in transcripts per million; TPM) using Kallisto 0.44.0 [64]. The resulting library was screened for peptide-encoding transcripts using (1) blast homology searches against the NCBI protein database filtered for vertebrate sequences and (2) blastx searches against an in-house built database of known amphibian skin peptides. Transcripts that returned positive blast hits (e-values < 10^−5^) were scanned for the presence of *N*-terminal signal peptides using SignalP 4.1 [65] and were visually checked for basic peptide cleavage sites (-KR-, -RR- and -RXXR-) to predict the full sequence of functional peptides. Predicted peptides were submitted to the online structure prediction tool PSIPRED v3.3 [31] to assess the likelihood of secondary structures.

To identify candidate transcripts encoding the two previously described *P. microps* peptides [20], the assembled transcript libraries were screened using blastx searches against a database containing the two peptides as well as all isoforms that differed in one amino acid (382 peptides in total). Since it is possible that these searches will not give positive hits due to short sequence of the peptides, we additionally used a text editor to screen a library of translated sequences using the two peptides as search strings. This library was obtained by translating all open-reading frames > 150 bp in the two *Phrynomantis* transcript libraries using the Trinity utility Transdecoder [27].

### 4.4. Peptidome Analyses

To confirm the actual secretion of predicted peptides in *P. microps*, 10 µL of diluted skin secretion sample was injected into a Waters Breeze analytical HPLC system coupled to a Micromass Q-Tof *micro* system. The HPLC is equipped with a Waters 2696 pump, Waters 2489 UV/visible detector (at a wavelength of 215 nm) and a Grace Vydac C18 column (15 cm × 2.1 mm × 3 µm) with a flow rate of 0.3 mL/min. Electrospray data were acquired by electrospray positive ionization mode (ESI+) scanning over the mass-to-charge ratio (m/z) scale from 100 to 3000 at a scan time of one second and a cone voltage of 38 V. Data collection and analysis were done with Masslynx software (Waters Co.).

### 4.5. Peptide Synthesis

Five of the predicted peptides were *de novo* synthesized using solid-phase technology (outsourced to Synpeptide, Shanghai, China) and delivered as HPLC-purified TFA salts (>95%). Peptide salts were stored as 5.12 mM stock solutions in 0.01% (V/V) acetic acid/0.2% (*m*/*V*) BSA at 4 °C until further use.

### 4.6. Antimicrobial and Cytotoxicity Assays

Peptides were tested for antimicrobial activity against the gram-negative species *Escherichia coli* (ATCC 25922) and the gram-positive species *Staphylococcus aureus* (ATCC 25923) and for cytotoxic activity towards intestinal epithelial cells using the human cancerous colonic cells (Caco-2) monolayer model. All assays were performed in triplicate. Antimicrobial activity against bacteria was measured by assessing the lowest peptide concentration at which no growth was detected in a series of twofold dilutions (minimum inhibitory concentration, MIC). Bacterial cultures (5 × 10^5^ colony forming units per ml) were prepared in Müller-Hinton broth and transferred to serial dilutions of peptides ranging from 512 µM down to 1 µM in 96-well polypropylene plates. After incubation at 37 °C for 18 h, growth of the cultures was visually checked.

The cytotoxic activity of peptides against Caco-2 cells was measured by quantifying the fraction of surviving cells after peptide treatment using a neutral red assay. Caco-2 cells were seeded at an initial concentration of 5 × 10^5^ cells/mL in Hank’s balanced salt solution medium with Mg^2+^ and Ca^2+^ (HBSS+) in polystyrene flat-bottom 96-well plates and incubated for 21 days (at 37 °C, 5% CO_2_ and 95% humidity) to grow morphologically differentiated monolayers of interconnected cells. Cytotoxic activity of peptides was verified by exposing the resulting monolayers for 15 min to a range of peptide concentrations between 512 and 0.5 µM. Five wells with cell medium only (HBSS+) and five wells containing 1% Triton-X solution were used as negative and positive controls, respectively. After 15 min, peptide solutions were removed and 200 µL neutral red solution (33 µg/mL) was added to each well. After two hours of incubation at 37 °C, the dye solution was removed and cells were rinsed three times with 200 µL HBSS+ solution. Wells were then incubated with 200 µL glacial acetic acid solution (glacial acetic acid: ethanol: water = 1:50:49) for 10 min in darkness. Finally, 150 µL medium from each well was transferred to an optically clear ELISA well-plate, after which the optical density (OD) was measured at 550 nm using a multiskan microplate photometer/ELISA reader (Thermo Scientific, Waltham, MA, USA). The cytotoxic activity for each peptide concentration, expressed as a percentage, was calculated as 100 × (OD_obs_ − OD_0_)/(OD_100_ − OD_0_), where OD_obs_ is the OD measured for the peptide concentration, OD_0_ is the average OD for the negative controls (0% cytotoxicity) and OD_100_ is the average OD after treatment with Triton-X (100% cytotoxicity).

### 4.7. Insecticidal Activity Experiments

Insecticidal activity was tested on larvae of the greater wax moth *Galleria mellonella* [66,67]. Sixth-instar larvae were obtained from a commercial source (Live Bait Shop, The Netherlands) and kept at 15°C to avoid cocoon formation. Only healthy larvae with a weight between 200 mg and 400 mg were used in the experiment. The experiment consisted of exposing randomly defined groups of larvae (n = 8) to one of the following treatments—(1) injection of 20 µL 6× diluted *P. microps* skin secretion (in mQ water; see 2.2), to assess whether the skin secretion as a whole has any insecticidal activity; (2) for each peptide, injection of 20 µL 1mM solution (dissolved in 0.01% acetic acid/0.2% BSA), to investigate the insecticidal activity of individual peptides; (3) injection of all peptides together (each at 1 mM) to test for a possible synergistic effect among the peptides; (4) injection of 20 µL of the 0.01% acetic acid/0.2% BSA solvent, to serve as a negative control and (5) no injection, to serve as background. Injections involved insertion of a 30 G needle in the last left proleg. After injection, larvae were placed on their backs in a 10 × 10 cm box to check for a reorientation response (turning of the body until the ventral side faced the bottom). A grid paper with 5 × 5 mm squares placed on the bottom of the box was used to measure crawling activity of the larvae at 10 min and 60 min after reorientation, quantified as the number of squares through which the head moved during a one-minute interval. Individuals were checked for dark discoloration of the skin (indicating the onset of dying) and responsiveness to tactile stimuli (indicating sedation or onset of death) before each measurement of crawling activity. We used generalized linear modelling as implemented in SPSS (version 24) to test for differences in crawling activity (counts/min) among treatments (treatment as main effect), using a negative binomial distribution and a Sidak post-hoc test. Differences in crawling activity were considered significant at *p* < 0.05.

### 4.8. Inflammation Assays

Spleen and bone marrow (tibia and femur) cells were obtained by homogenizing the organs of three CO_2_ euthanized eight-weeks-old female C57Bl/6 mice in 10 mL RPMI medium, passing the suspension through a 40 mm pore filter (removing crude debris) and centrifugation (7 min, 398 g, 4 °C). The pellet was treated with erythrocyte-lysis buffer to remove red blood cells from the mixture, following centrifugation (7 min, 398 g, 4 °C) and resuspending of the pellet in 2–5 mL RPMI medium plus 5% Fetal Calf serum (FCS). Cells were counted to bring them at 2.5 × 10^6^ cells/mL in RPMI-1640 medium with 10% FCS plus 1% nonessential amino acid mixture (Sigma-Aldrich), glutamate and streptomycin and transferred to polystyrene cell culture well plates (180 μL per well). After adding 20 µL of 1mM peptide solution (resulting in a 100 µM peptide concentration), cell cultures were incubated for two hours in a humidified incubator (37 °C, 5% CO_2_). Negative control wells were created by adding 20 µL of 0.01% acetic acid/0.2% BSA (solvent without peptide) and positive control wells were created by adding 20 µL of 1 µg/mL *E. coli* lipopolysaccharide/endotoxin (LPS). After incubation, all cell cultures media were collected from the wells and submitted to different enzyme-linked immunosorbent assays (ELISA), measuring the amounts of released TNF-α (R&D systems, USA), NO (Nitric oxide; R&D systems, USA), INF-γ (Pharmingen Inc. Taiwan) and IL-6 (Pharmigen Inc. Taiwan). All ELISA were performed according to the suppliers’ manuals, with all optical densities (OD) measured at the recommended wavelength using a multiskan microplate photometer/ELISA reader (Thermo Scientific, USA).

### 4.9. Phylogenetic Analyses

Spleen To reconstruct the evolutionary history of newly discovered peptide-encoding transcripts, they were aligned with 102 FSAP transcript sequences retrieved from GenBank. These sequences represent the majority of previously described FSAP peptide families across various neobatrachian taxa. The alignment was created using the E-INS-I algorithm implemented in MAFFT 7.407 [68]. Phylogenetic relationships were estimated with MrBayes 3.2.6 [69] using a general time-reversible model of DNA substitution with gamma correction for among-site rate heterogeneity and an estimated proportion of invariable sites (GTR + G + I). Two runs of four Markov chain Monte Carlo chains each were executed in parallel for 20 million generations, with a sampling interval of 1000 generations and a burning of two million generations. Convergence of the parallel runs was confirmed by split frequency standard deviations (<0.01) and potential scale reduction factors (approximating 1.0) for all model parameters using Tracer 1.7 [70], by verifying if the runs had reached effective sampling sizes > 200 for all model parameters.

## 5. Conclusions

The present study provides an overview of the repertoires of skin-secreted peptides and proteins of *Phrynomantis bifasciatus* and *P. microps*, two poisonous species belonging to the poorly studied frog family Microhylidae. Our results suggest a major structural diversity and high abundance of serine protease inhibitors and of ancient vertebrate antimicrobial peptide and protein families. In addition, we demonstrate that the frog skin active peptide (FSAP) superfamily, a widespread group of evolutionarily related bioactive peptides has been evolutionarily preserved in at least one microhylid lineage. Their deviant structural diversity and physicochemical properties compared to related peptides in other frogs warrants their classification in a new peptide family (phrynomantins). Despite the current lack of a known biological function, the discovery of phrynomantins encourages research on the skin secretion of other microhylid frogs. In addition, our study could inspire the exploration of similarly understudied frog neobatrachian families, like the comparably species-rich and ecologically diverse Centrolenidae, Craugastoridae, Hemiphractidae and Arthroleptidae. Broadening the research to encompass a wider range of frog families would not only further elucidate the origin and evolution of the FSAP superfamily as a fascinating chemical defence adaptation but also reveal new peptides with previously unknown physicochemical properties and bioactivities.

## Figures and Tables

**Table 1:** Protein families encoded by recovered transcripts in both *Phrynomantis* species, including estimated transcript diversity (number of assembled transcript contigs) and estimated total expression level (sum of transcripts per million (TPM) of contigs representing the same gene/protein family).

	*Phrynomantis bifasciatus*		*Phrynomantis microps*	
	Number of Transcript Contigs	∑TPM ^1^	Number of Transcript Contigs	∑TPM ^1^
**Frog Skin Active Peptide (FSAP) superfamily**	10	13,437.3	7	12,636.8
**Antimicrobial peptides/protein families**				
Bactericidal permeability-increasing protein (BPI)	48	992.4	38	479.6
Beta-defensin	1	1.7	1	3.5
Cathelicidin	6	5.1	5	4.8
C-type lysozyme	9	127.4	6	578.9
G-type lysozyme	12	5.6	2	5.0
**Serine protease inhibitor families**				
Kazal-like	1	408.4	4	534.7
Kunitz/Bovine pancreatic trypsin inhibitor (BPTI)	20	941.0	15	423.4
Serpin-type	60	773.2	60	2119.8
Trypsin inhibitor-like (TIL)	39	10,424.7	45	4252.5
Whey acidic protein (WAP)	26	506.0	20	825.2
**Hormone/neuropeptide-like peptide families**				
Angiotensin	1	1.9	0	0
Bradykinin (kininogen)	2	2.9	1	7.2
Gastrin	1	0.2	0	0
Glucagon	1	1.0	0	0
Natriuretic peptide	2	2.2	1	3.3
Neurotensin/Neuromedin N	1	0.7	0	0
Secretin	1	5.0	1	1.4
Secretogranin	11	35.6	8	70.0
Tachykinin	0	0	2	7.1

^1^ Calculated by summing the expression levels (in transcripts per million (TPM)) of all transcript contigs identified as encoding members of the same protein family.

**Table 2:** Physicochemical and structural properties of the predicted phrynomantins.

Peptide	Sequence ^1^	Length	MW (Da) ^3^	Net charge ^4^	GRAVY ^5^	Helicity (%) ^6^
phrynomantin-1Ba	GLVTNLLSSVR	11	1158.4	1	0.84	72.7
phrynomantin-1Bb	GLVPDLDLPVDL	12	1265.5	−3	0.79	0
phrynomantin-1Bc	GIVNNLLSTVL	11	1142.4	0	1.40	81.8
phrynomantin-1Bd	GPVFDYLSQVYPVR	14	1639.9	0	0.05	50
phrynomantin-1Be	GLVKDILSLDVL	12	1284.5	−1	1.33	66.7
phrynomantin-1Bf	GLVPDLDLPVDLPV	14	1461.7	−3	0.86	0
phrynomantin-1Ma	GLVKNQDLPVDLAAVF	16	1699.0	−1	0.66	12.5
phrynomantin-1Mb	GLVKNLNLPVDVPVDL ^2^	16	1705.0	−1	0.66	0
phrynomantin-1Mc	GLVKDLNLPVDVPVDLPV	18	1902.2	−2	0.73	0
phrynomantin-1Md	GLVKDLLSLDV^2^	11	1171.4	−1	1.05	63.6
phrynomantin-2Ba	DYEAVSL	7	795.8	−2	0.10	0
phrynomantin-2Ma	DYEPASL	7	793.8	−2	−0.73	0
phrynomantin-3Ba	SEWPPVRGDNGEYDVEL	17	1962.0	−4	−1.19	0
phrynomantin-3Ma	SEWPPVRGDAGEYDVEL	17	1919.0	−4	−0.88	0
phrynomantin-3Mb	AEWRLL	6	786.9	0	0.08	0
phrynomantin-3Mc	AEWRLLKNa	8	1028.2	2	−0.86	0
phrynomantin-4Ba	NAASRLYTPYSPTK	14	1568.7	2	−0.95	0
phrynomantin-4Bb	NARNFNSFDPFNTSD	15	1745.8	−1	−1.28	0
phrynomantin-4Ma	NTENFFNPFNPFV^2^	13	1586.7	−1	−0.46	0

^1^ Residues predicted to be involved in helical structure are underlined; a, C-terminal amidation. ^2^ Presence in secretion confirmed by LC-MS. ^3^ MW, Molecular weight. ^4^ at pH = 7.0. ^5^ GRAVY, Grand average of hydropathy. ^6^ As predicted using Psipred [31].
